# Predictors and prevalence of lateral supraclavicular lymph node metastases on [^18^F]FDG PET/CT in patients with locally advanced, or multifocal invasive ductal carcinoma of breast: potential impact on adjuvant radiotherapy targets

**DOI:** 10.3389/fonc.2026.1916551

**Published:** 2026-08-04

**Authors:** Ur Metser, Seyed Ali Mirshahvalad, Vanessa Murad, Patrick Veit-Haibach, Ezra Han

**Affiliations:** 1 University Medical Imaging Toronto; Joint Department of Medical Imaging: University Health Network, Sinai Health Systems & Women’s College Hospital, Toronto, ON, Canada; 2 Department of Medical Imaging, University of Toronto, Toronto, ON, Canada; 3 Department of Medical Imaging, Health Sciences North, Northern Ontario School of Medicine University, Sudbury, ON, Canada; 4 Radiation Medicine Program, Princess Margaret Cancer Centre, University Health Network, Toronto, ON, Canada; 5 Department of Radiation Oncology, University of Toronto, Toronto, ON, Canada

**Keywords:** breast malignancy, N stage, node-positive, radiation treatment planning, regional nodes, supraclavicular field

## Abstract

**Purpose:**

There is variability in the coverage of the lateral supraclavicular fossa in adjuvant radiotherapy targets in breast cancer patients. The purpose of this study was to evaluate the prevalence and predictors of lateral supraclavicular lymph node metastasis (LSCLNM) in patients with advanced or multifocal breast cancer on [^18^F]FDG-PET/CT.

**Materials and Methods:**

Invasive ductal breast carcinoma patients who were referred for [^18^F]FDG-PET/CT were included. Primary tumor location, [^18^F]FDG-PET parameters, extent of regional nodal disease (including presence or absence of LSCLNM), and distant metastasis status were recorded. Associations between these parameters and presence of LSCLNM were tested.

**Results:**

There were 243 patients evaluated, of whom 23 had bilateral disease. Of the 266 primary breast tumors evaluated, 19 (7.1%) cases had ipsilateral LSCLNM. None of the patients with locoregional nodal metastasis limited to axillary levels 1–2 had LSCLNM, whereas 8.7%, 12.8% and 52.2% of cases with axillary nodal metastasis up to level III, axillary nodal plus internal mammary nodal metastases, and medial supraclavicular lymph node metastasis (MSCLNM) showed LSCLNM, respectively. Further associations with LSCLNM included metabolic tumor volume (MTV), tumor location in the upper outer quadrant and presence of distant metastasis. Following multivariate analysis, only MTV and the presence of axillary level III nodal metastasis and MSCLNM remained statistically significant (p of 0.043, 0.011 and <0.001, respectively), suggesting their independence.

**Conclusions:**

LSCLNM was identified in a minority of patients with breast cancer undergoing staging with [^18^F]FDG-PET/CT, but its likelihood increased substantially with more extensive regional nodal disease. Specialists reporting PET scans should be aware of the clinical implications of this finding and ensure to report the nodal spread in detail. Our data suggest that in patients with PET-positive high-level nodal disease, inclusion of the LSCLN should be considered, based on individual risk features including tumor location and primary tumor burden.

## Introduction

The presence of locoregional nodal metastases in breast cancer impacts prognosis and patient management. Breast cancer usually spreads in a predictable pattern along the axilla (axillary level I →→ II → III), and then to supraclavicular nodes (1–3). Internal mammary nodal metastases may follow a separate pathway and are most often encountered in patients with primary tumors located in the inner quadrants of the breast (4). In a minority of patients (2-7%), there may be skip metastases to non-contiguous nodal basins (5). Regional nodal irradiation for patients with axillary node-positive breast cancer after primary surgery has been shown to improve disease-free survival, decrease the rate of distant metastases and improve overall cancer-specific mortality (6). In addition to the axillary nodal basins, supraclavicular nodes are an integral part of regional nodal irradiation in these patients. However, the clinical target volumes for radiotherapy in the supraclavicular nodal basin vary widely (7–9). The Radiation Therapy Oncology Group (RTOG) and European Society for Radiotherapy and Oncology (ESTRO) breast contouring atlases define the medial supraclavicular fossa nodal basin (MSCLN; neck level IV), effectively excluding the lateral supraclavicular fossa nodal basin (LSCLN; neck level Vb) while others include it as an optional volume in addition to the MSCLN (10–12).

A prior randomized trial has shown that in addition to more frequent upstaging to stage IV, [^18^F]FDG PET/CT detects regional nodal metastases more often than conventional staging procedures (22.7% vs 7.1/%, respectively) in invasive ductal breast carcinoma (IDC; also known as breast cancer of no special type) (13). With the increasing use of [^18^F]FDG PET to stage patients with locally advanced breast cancer (LABC) or those with equivocal findings for advanced disease on conventional workup, more gross locoregional nodal metastases will be identified, including in the supraclavicular fossae. The purpose of this analysis is to determine the prevalence and predictors of LSCLN metastases on [^18^F]FDG PET/CT in IDC patients.

## Materials and methods

### Study population

In this institutional ethics review board-approved retrospective study, consecutive patients referred for staging of IDC from December 2016 to March 2024 were included. Informed consent was waived. Patients included were staged with mammography, breast sonography ± MR breast as well as [^18^F]FDG PET/CT. Exclusion criteria included histology of invasive lobular breast cancer, the presence of a second primary malignancy, and initiation of systemic therapy or surgery prior to [^18^F]FDG PET/CT.

### [^18^F]FDG PET/CT imaging protocol

Approximately 60 minutes after intravenous injection of 300–400 MBq (4–5 MBq/kg) of [^18^F]FDG, PET/CT imaging was acquired from the top of the skull to the upper thighs on a Siemens mCT40 PET/CT scanner (Siemens Healthineers, Erlangen, Germany) or on an in-line Biograph PET/CT scanner (Siemens Healthineers, Erlangen, Germany). Same vendor scanners were harmonised (EARL-compliant) to minimise differences in imaging-derived parameters. Continuous Bed Movement (CBM) was used whereby time per bed is replaced with corresponding bed speed, typically 1.0 mm/sec over area of interest. CT was used for attenuation correction and localization using the following scan parameters: tube voltage 120 kV peak, 40–150 mAs, collimation 2 mm, rotation time 0.8 s, feed/rotation 8.4 mm. Images with slice thickness of 5 mm were reconstructed in the axial plane using a soft tissue kernel.

### Image analysis

Two expert readers (UM and SAM with 23 and 5 years of experience) reviewed [^18^F]FDG PET/CT images independently. Readers were blinded to the clinical and pathological stage and findings on other imaging modalities. Detailed staging data were collected, including locoregional node status. In terms of primary tumor characteristics, tumor location in the breast (using 4 quadrants) and [^18^F]FDG PET-derived parameters, including SUVmax, metabolic tumor volume (MTV) and total lesion glycolysis (TLG), were extracted. Tumors were assessed and segmented using Hermes workstation (Hermes Medical Solutions, Montreal, Canada). Also, regional nodal metastases were rated as positive, negative, or equivocal. The regions included ipsilateral axillary level I, axillary level II, axillary level III, internal mammary, neck level IV (MSCLN) and neck level Vb (LSCLN). The final interpretations were made following a consensus meeting to resolve discrepancies. Notably, no SUVmax cut-off was used for interpretation and both subjective assessment of metabolic activity (above blood pool activity) along with CT appearance (e.g. reactive morphology) were considered for qualitative decision-making.

### Statistical analysis

Descriptive statistics are reported as counts (percentages) and mean (standard deviation [SD]) for categorical and continuous variables, respectively. The 95% confidence intervals (CIs) for proportions were also estimated. There were no missing data. Associations between each nodal station and ipsilateral neck level Vb were tested with logistic regression. For this purpose and the next analyses, equivocal nodes were treated as negative to have a more robust methodology. For the ordinal “extent of nodal metastasis” variable, we assessed the N-stage trend as per the American Joint Committee on Cancer v.8 guidelines (14), by fitting a univariable logistic regression with the coded extent and reported the odds ratio per one-level increase; in this regard, we defined the order as axillary level I/II nodal metastasis alone (to reflect N1/N2a); internal mammary nodal metastasis in isolation (to reflect N2b); axillary level III nodal metastasis (to reflect N3a); internal mammary nodal metastasis with axillary nodal metastases (to reflect N3b); MSCLN metastasis (to reflect N3c). Since internal mammary nodal metastasis in isolation is rare (3) (we only had 2 cases [<1%]) and may suggest an alternate lymphatic pathway to axillary/supraclavicular spread (none of our 2 cases had LSCLN metastasis), for the purpose of our analysis and after excluding LSCLN metastasis, we used increasing nodal stage as N1/2a → N3a → N3b → N3c to assess for increased risk of LSCLN metastasis.

Moreover, the association between other factors, including primary tumour characteristics and presence of distant metastasis, with LSCLN metastasis was assessed. In this regard, for primary tumour-derived [^18^F]FDG PET parameters (SUVmax, MTV and TLG), variables were initially assessed with their continuous nature, and then, the significantly associated variables were dichotomized (the best cut-off was determined by receiver operating characteristic curve analysis using Youden’s index) and further assessed by univariate and multivariate regression analyses, since the high collinearity between variables is well-established (TLG is a product of MTV times SUVmean; MTV was a more robust predictor and used in the final modelling).

For the final comprehensive analysis (including primary tumor characteristics, regional nodal status and distant metastasis), the site/quadrant of primary tumor, primary tumor-derived MTV, site of regional nodal metastasis (presence of axillary level III, internal mammary, and MSCLN metastasis), and presence of distant metastasis were screened for the multivariate assessment. The final multivariate analysis was done following Least Absolute Shrinkage and Selection Operator (LASSO) as a penalized linear regression method to prevent overfitting, considering the low number of events relative to the available significant variables from earlier analyses. Notably, all screened predictors (n = 6) were retained following LASSO selection in the final multivariate regression model. Furthermore, because some patients (n = 23) had bilateral disease in our cohort, sensitivity analyses (using generalized estimating equations) were conducted at this stage to account for within-patient clustering and generate more realistic adjusted estimates. Analyses were conducted in IBM SPSS Statistics (version 29). A two-sided p-value <0.05 was considered statistically significant.

## Results

### Study population characteristics

**Figure 1:**
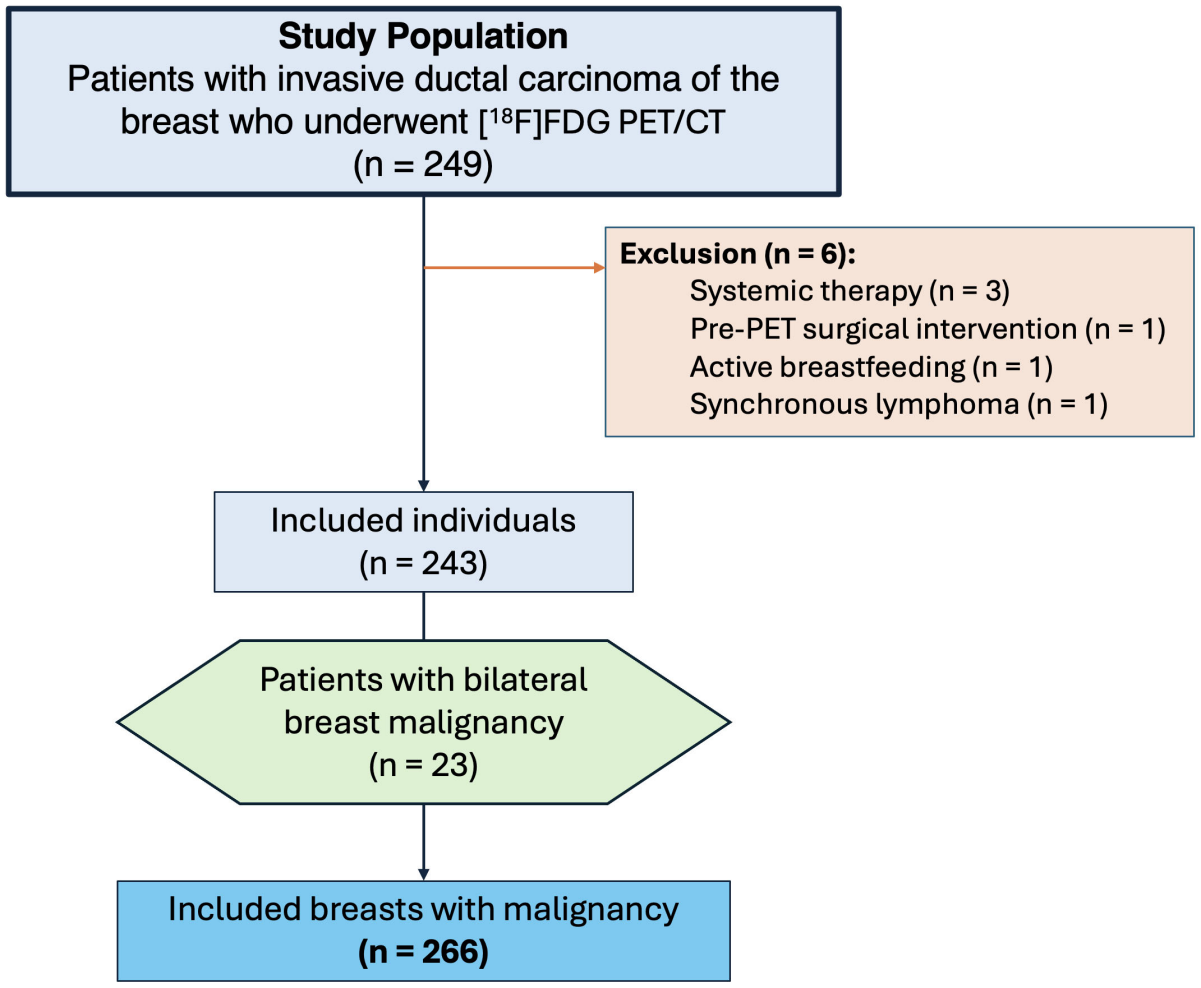
Patient selection flowchart.

There were 249 female patients who were assessed for eligibility. The patient flowchart is provided in **Figure 1**. Six patients were excluded due to initiation of systemic therapy (n = 3), post-operative changes (n = 1), patient lactation (n = 1), and synchronous malignancy (n = 1). The patient selection flowchart is provided in **Figure 1**. The final study cohort comprised 243 patients with IDC; 23 of whom had bilateral disease. Patients were referred for [^18^F]FDG PET/CT for staging of LABC (n=204), multifocal or bilateral disease (n=23 and 4, respectively), staging of recurrent breast cancer (n=4), or equivocal findings for distant metastatic disease on CT or bone scintigraphy (n=8).

For each patient, the 6 regional nodal stations were assessed. Overall, 1596 nodal stations (266 x 6 regional nodal basins) were evaluated for the presence of metastatic locoregional nodes. Patient demographics including pre-PET clinical stage, location of malignancy, [^18^F]FDG PET semiquantitative measures, distribution of nodal metastases and presence or absence of distant metastases are shown in **Table 1**. Of the 266 primary breast tumors evaluated, 19 (7.1%) cases had ipsilateral LSCLN metastasis.

**Table 1:** Patients’ characteristics (for a total of 266 breasts with invasive ductal carcinoma).

Parameter	Frequency (%)	Mean (SD; range: min-max)
Age (years)		55.1 (14.4; 25-93)
Pre [^18^F]FDG PET/CT stage		
Stage IA Stage IIA Stage IIB Stage IIIA Stage IIIB Stage IIIC NA*	12 (4.5%) 21 (7.9%) 149 (56.0%) 57 (21.5%) 11 (4.1%) 8 (3.0%) 8 (3.0%)	
Primary tumor: location		
Upper outer quadrant ** Any lateral quadrant Only medial quadrants	156 (58.6%) 201 (75.6%) 65 (24.4%)	
Primary tumor SUVmax		12.57 (7.69; 2.0-47.7)
Primary tumor MTV (mL)		29.13 (76.98; 0.02-993.14)
Primary tumor TLG		155.82 (294.79; 0.04-2230.00)
Regional nodal metastasis		
Yes No Equivocal	209 (78.6%) 36 (13.5%) 21 (7.9%)	
Site of regional nodal metastasis		
Axillary level 1		
Yes No Equivocal	202 (75.9%) 43 (16.2%) 21 (7.9%)	
Axillary level 2		
Yes No Equivocal	136 (51.2%) 123 (46.2%) 7 (2.6%)	
Axillary level 3.		
Yes No Equivocal	42 (15.7%) 222 (83.5%) 2 (0.8%)	
Internal mammary		
Yes No Equivocal	52 (19.5%) 208 (78.2%) 6 (2.3%)	
MSCLN		
Yes No Equivocal	23 (8.6%) 242 (91.0%) 1 (0.4%)	
LSCLN		
Yes No Equivocal	19 (7.1%) 245 (92.1%) 2 (0.8%)	
Distant metastasis		
Yes No Equivocal	51 (19.2%) 206 (77.4%) 9 (3.4%)	

*Histologically proven IDC in the contralateral breast with contralateral metabolically active mass on [^18^F]FDG PET/CT subsequently shown to be synchronous IDC.

**Involvement of upper outer quadrant with or without involvement of other quadrants.

MTV, metabolic tumor volume; TLG, total lesion glycolysis

### Extent of regional nodal metastasis

Presence of LSCLN metastases as per presence of other locoregional nodal metastases is presented in Supplemental **Supplementary Table 1**. Extent of regional nodal metastasis, including nodal metastasis limited to axillary levels I and/or II (n = 122), axillary nodal metastasis up to level III (n = 23), internal mammary nodal metastasis with axillary nodal metastases (n = 39), and presence of MSCLN metastasis (n = 23), was assessed to evaluate the association of the extent of regional nodal metastasis with LSCLN metastasis. None of the patients with locoregional nodal metastasis limited to axillary level I and/or II had LSCLN metastasis. However, 8.7% (95%CI: 2.4%-26.8%), 12.8% (95%CI: 5.6%-26.7%) and 52.2% (95%CI: 33.0%-70.8%) of cases with axillary nodal metastasis up to level III, axillary nodal metastasis plus internal mammary nodal metastasis, and MSCLN metastasis showed LSCLN metastasis, respectively. Based on logistic regression, a one-level increase in extent of nodal metastatic disease was associated with 6-fold higher odds of LSCLN metastasis (odds ratio = 6.00, 95% CI 2.93–12.27; p < 0.001).

Association of LSCLN metastases with other nodal sites is presented in **Table 2**. The presence of nodal metastasis in axillary level III, internal mammary, and MSCLN are independent predictors of the presence of LSCLN metastasis.

**Table 2:** Logistic regression results for the association of the site of regional nodal metastasis with ipsilateral lateral supraclavicular lymph node (LSCLN) metastasis.

Distal nodal extent	LSCLN metastasis		
	Odds ratio	95%CI	p-value
Univariate analysis			
Axillary level 1 nodal metastasis	–	–	-*
Axillary level 2 nodal metastasis	–	–	-*
Axillary level 3 nodal metastasis	12.40	4.53-33.95	<0.001
Internal mammary nodal metastasis	6.91	2.62-18.23	<0.001
MSCLN metastasis	36.78	12.11-111.72	<0.001
Multivariate analysis			
Axillary level 3 nodal metastasis	8.53	2.48-29.33	<0.001
Internal mammary nodal metastasis	3.85	1.14-13.00	0.030
MSCLN metastasis	24.87	7.07-87.54	<0.001

*Not estimable due to complete separation.

### Primary tumor characteristics and distant metastasis

The association of the primary tumor characteristics and presence of distant metastasis with LSCLN metastasis was also assessed. Tumor location in the upper outer quadrant was significantly associated with LSCLN metastasis (odds ratio of 6.60, 1.49-29.20; p = 0.013), while malignancy in the outer quadrants in general was not (p = 0.997). The association of primary tumor-derived [^18^F]FDG PET parameters are provided in **Table 3**. As seen, among the studied parameters, MTV of the primary tumor was an independent predictor of LSCLN nodal metastasis. Notably, the area under the curve for SUVmax, MTV and TLG were 0.68, 0.81 and 0.81, respectively, and the optimal cut-offs for these parameters were set at 11, 22.5, and 127, respectively. Furthermore, the presence of distant metastasis was also strongly associated with presence of LSCLN metastases with an odds ratio of 8.87 (3.29-23.92; p <0.001).

**Table 3:** Logistic regression results for the association of the primary tumour-derived [^18^F]FDG PET parameters with ipsilateral neck level Vb nodal (lateral supraclavicular lymph node [LSCLN]) metastasis.

Primary tumor characteristic	LSCLN metastasis		
	Odds ratio	95%CI	p-value
Univariate analysis for continuous parameters			
Primary tumor SUVmax	1.06	1.01-1.11	0.035
Primary tumor MTV	1.00	1.00-1.01	0.044
Primary tumor TLG	1.00	1.00-1.00	0.001
Univariate analysis after dichotomizing parameters			
Primary tumor SUVmax	5.89	1.67-20.7	0.006
Primary tumor MTV	14.69	4.144-52.06	<0.001
Primary tumor TLG	4.67	0.88-24.89	0.071
Multivariate analysis for binary parameters			
Primary tumor SUVmax	2.34	0.60-9.13	0.222
Primary tumor MTV	10.69	2.81-40.69	<0.001

MTV, metabolic tumor volume; TLG, total lesion glycolysis.

### Final multivariate regression model

Results of a multivariate analysis of all independent parameters (six earlier-identified parameters; retained following LASSO regression) predicting LSCLN metastases can be found in **Table 4**, using the within-patient clustering. MTV, axillary level III nodal metastasis, and MSCLN metastasis were independently associated with increased risk of LSCLN metastasis (p < 0.05).

**Table 4:** Multivariate logistic regression results for the association of significant site of primary disease (upper outer quadrant), significant tumour-derived [^18^F]FDG PET parameter (metabolic tumour volume), significant sites of regional nodal metastasis (axillary level 3 and internal mammary chain), and presence of distant metastasis with the presence of ipsilateral lateral supraclavicular lymph node (LSCLN) metastasis.

Multivariate analysis	LSCLN metastasis		
	Adjusted odds ratio	95%CI	p-value
Upper outer quadrant primary tumor	3.49	0.50-24.44	0.208
Primary tumor MTV	4.90	1.05-22.82	0.043
Axillary level 3 nodal metastasis	6.65	1.55-28.61	0.011
Internal mammary nodal metastasis	2.32	0.51-10.46	0.275
MSCLN metastasis	24.37	5.81-102.28	<0.001
Distant metastasis	1.83	0.44-7.67	0.406

MTV, metabolic tumor volume.

## Discussion

In this retrospective cohort of patients with invasive ductal breast cancer staged with [^18^F]FDG PET/CT (most of whom had LABC), regional/ipsilateral LSCLN metastases were identified in 7.1% of individuals. The probability of LSCLN involvement was strongly related to the extent and distribution of regional nodal disease. Notably, no LSCLN metastases were identified when nodal disease was absent or limited to axillary levels I or II. In contrast, LSCLN involvement was seen in 8.7%, 12.8% and 52.2% of cases with axillary nodal metastasis up to level III, axillary nodal metastasis plus internal mammary nodal metastasis, and presence of MSCLN metastasis, respectively. On multivariable analysis, MSCLN involvement was the strongest predictor of LSCLN metastasis, with axillary level III involvement and primary tumor MTV also remaining independently associated with LSCLN disease.

These findings are consistent with the expected stepwise pattern of lymphatic spread in breast cancer, while also highlighting the importance of assessing the full extent of nodal disease in patients with high-risk presentations. Breast cancer commonly spreads from the axillary levels I → II → III and subsequently to the supraclavicular fossa, although internal mammary involvement and skip metastases can occur (5). In this regard, one prior prospective study (3) including 509 patients with *de novo* metastatic breast cancer showed that most patients with level II nodal metastases have level I metastases and most those with level III have level II metastases; among those with axillary involvement, less than 1% exhibited isolated level II or III disease without involvement of level I. Additionally, no patients had isolated supraclavicular or internal mammary disease without axillary involvement. Other previously published studies also reported a considerably low incidence of isolated ipsilateral supraclavicular nodal metastasis in breast cancer patients, being less than 5% (15, 16). In this context, the absence of LSCLN metastases in our study among patients with disease limited to axillary levels I–II or with isolated internal mammary metastasis is clinically reassuring. Conversely, the high rate of LSCLN involvement among patients with MSCLN metastases suggests that disease in the medial supraclavicular fossa may be an important marker for more extensive supraclavicular nodal spread laterally/posteriorly.

The clinical relevance of these findings relates directly to radiotherapy target volume selection. Regional nodal irradiation has been shown to reduce breast cancer recurrence and improve disease-related outcomes in selected patients with node-positive or high-risk breast cancer (17). However, the optimal extent of supraclavicular nodal coverage remains uncertain. Current contouring atlases differ in how they define the supraclavicular nodal clinical target volume, with commonly used RTOG and ESTRO-based approaches generally emphasizing the medial supraclavicular fossa/neck level IV and not routinely including the lateral supraclavicular fossa/neck level Vb/posterior neck. The present study addresses this gap by describing the prevalence of [^18^F]FDG PET-positive LSCLN metastases and identifying clinical and imaging features associated with their presence.

[^18^F]FDG PET is an accurate modality for the assessment of metastatic nodal spread in breast patients with IDC (18), including supraclavicular nodal metastasis evaluation (19). In node-negative patients, large trials (20) have shown no benefit in supraclavicular irradiation in terms of overall survival, disease-free survival, distant metastasis-free survival and breast cancer recurrence. On the other side of the spectrum, for patients with gross supraclavicular/LSCLN disease, inclusion of this region in the gross tumor volume is straightforward. However, considering our findings, patients with [^18^F]FDG PET-positive MSCLN disease, axillary level III disease, or large metabolically active primary tumors may represent a subgroup in whom careful review of the lateral supraclavicular fossa is warranted. Importantly, in these patients, lateral supraclavicular nodes with minimal metabolic activity can also be of concern (21, 22). Furthermore, it was shown that along with large tumor size, HER2- subtype was also significantly correlated with the presence of LSCLN metastasis (23), which was not assessed in our study. Therefore, in addition to variables assessed in the current manuscript, the histopathological/molecular subtype of breast cancer may have a role in predicting nodal disease spread as well. Prior studies have developed nomograms to predict regional lymph node metastases in various malignancies including breast cancer (24–26). Future studies may assist in developing and validating a nomogram to predict the risk of LSCLN metastases, to help select patients that would best benefit from inclusion of the LSCLN basin in the radiotherapy target volume.

In clinical practice, in the context of node-positive disease, routine standard care treats all undissected lymph node regions including the axilla (levels 1-3), MSCLN and the internal mammary chain. Within this pattern of care, taking into account our data, it stands to reason that if the MSCLN warrant treatment, the LSCLN do as well. This question is of increasing importance as modern radiotherapy shifts from historical field-based plans towards inverse planning techniques sculpted to treat defined volumes (27). In the 2D/3D era, supraclavicular fields were determined by bony landmarks, typically using the cricoid cartilage and other bony landmarks to define the radiation field borders. These large fields incidentally irradiated the LSCLN/posterior neck and the cranial aspects of the neck through the beam arrangements, even if not intending to do so. Modern inverse planning (IMRT/VMAT) techniques specifically sculpt the dose to stay conformal to the contoured target. If the LSCLN/posterior neck is not contoured, the algorithm will not attempt to cover the region, delivering little dose to these regions that previously received prophylactic coverage by the nature of the beam arrangements. Therefore, in the context of modern radiation planning, understanding which patients are most likely to have LSCLN involvement is important for maintaining adequate nodal coverage while avoiding unnecessary expansion of treatment volumes.

The historical limitations of standard contouring guidelines are further highlighted by 3D recurrence-mapping studies, which demonstrate a pattern of geographic miss in the posterolateral supraclavicular region. The Radiotherapy Comparative Effectiveness (RADCOMP) Consortium breast atlas was developed in 2016 as a standardized reference for an ongoing Phase III clinical trial comparing proton and photon therapies (12, 28). Recognizing that highly conformal photon techniques (and proton therapy, which lacks exit dose) remove the broad bath of historical radiation fields, the RADCOMP committee defined an optional “Posterior Neck” clinical target volume (encompassing Neck Level Vb) bounded anteriorly by the medial supraclavicular volume and posteriorly by the trapezius muscle. The oncological relevance of this optional volume has been validated by several retrospective mapping analyses of regional failures. DeSelm et al. demonstrated that most supraclavicular recurrences falling outside standard guidelines, specifically 67% of RTOG and 89% of ESTRO “misses” were localized precisely within this posterolateral posterior neck zone (29). Similarly, in a PET/CT-based recurrence-mapping study by Beaton et al., standard RTOG and ESTRO contours provided complete per-patient coverage of regional recurrences in only 38% and 57% of patients, respectively (30). Notably, the addition of the RADCOMP Posterior Neck volume to these guidelines increased complete coverage to 48% for RTOG and 70% for ESTRO, underscoring that this posterolateral space represents a reservoir for subclinical disease in high-risk patients, consistent with the findings in our study.

The ongoing SUCLANODE trial is directly addressing this uncertainty by comparing medial supraclavicular irradiation with medial plus posterolateral/entire supraclavicular irradiation in high-risk breast cancer patients (31). Until prospective outcome data are available, the present study provides imaging-based evidence to inform individualized treatment planning.

This study has limitations. It is a retrospective single-institution analysis and included patients referred for [^18^F]FDG PET/CT, many of whom had LABC or otherwise high-risk disease. Therefore, the prevalence of LSCLN metastases observed here should not be generalized to all patients with early-stage breast cancer. In addition, LSCLN metastases were identified by [^18^F]FDG PET/CT interpretation rather than systematic pathologic confirmation. However, image review was performed independently by experienced readers blinded to clinical and pathologic staging information, with consensus resolution of discrepancies. Second, this study included only patients with invasive ductal carcinoma of the breast. The biology of invasive ductal carcinoma and lobular carcinoma of breast differs. Lobular carcinomas show a discohesive single-file growth patterns, resulting in suboptimal visualization on imaging, including on [^18^F]FDG PET (32), limiting assessment of nodal metastases. As such, these results may not be applicable to patients with invasive lobular carcinoma of the breast. Finally, this analysis describes the prevalence and predictors of LSCLN involvement at staging but does not evaluate patterns of recurrence or clinical outcomes after different radiotherapy target volume approaches.

In summary, LSCLN metastases were identified in a minority of patients with IDC undergoing staging with [^18^F]FDG PET/CT, but the likelihood of LSCLN involvement increased substantially with more advanced regional nodal disease, particularly in the presence of MSCLN metastases. These findings suggest that routine inclusion of the LSCLN is unlikely to be necessary for patients without regional nodal disease or with disease limited to lower axillary nodal levels. However, in patients with [^18^F]FDG PET-positive high-level nodal disease, particularly MSCLN or axillary level III involvement, inclusion of the LSCLN appears reasonable and may be tailored based on individual risk features such as extent of nodal disease, primary tumor burden, and tumor location. It may also be reasonable to include the LSCLN when the risk is sufficient to justify treatment of the MSCLN, at least until prospective data clarify whether this additional coverage improves outcomes. As modern radiation techniques increasingly conform dose to explicitly contoured targets, these data may help guide a balanced approach to supraclavicular target volume selection that preserves adequate coverage of nodal regions at meaningful risk while avoiding unnecessary treatment-related side effects. Also, specialists reporting PET scans should be aware of the clinical implications of these findings and ensure to report the nodal spread in detail.

## Data Availability

The raw data supporting the conclusions of this article will be made available by the authors, without undue reservation.
