# Supplementary material for: Predictors and prevalence of lateral supraclavicular lymph node metastases on [18F]FDG PET/CT in patients with locally advanced, or multifocal invasive ductal carcinoma of breast: potential impact on adjuvant radiotherapy targets
Source: Front Oncol. 2026 Aug 4;16:1916551. doi: 10.3389/fonc.2026.1916551 (PMC13481314; doi:10.3389/fonc.2026.1916551)
Supplement: Supplementary file 1 [file Table1.pdf]

SUPPLEMENTAL TABLE

Table S1. Prevalence of lateral supraclavicular lymph node (neck level Vb) metastasis considering the presence of regional nodal metastasis in other sites. Data are presented as frequency (%).

Site	Level Vb metastasis	
	Yes	No
Axillary level 1 nodal metastasis		
Yes	19 (7.1%)	183 (68.8%)
No	0 (0.0%)	64 (24.1%)
Axillary level 2 nodal metastasis		
Yes	19 (7.1%)	117 (44.0%)
No	0 (0.0%)	130 (48.9%)
Axillary level 3 nodal metastasis		
Yes	12 (4.5%)	30 (11.3%)
No	7 (2.6%)	217 (81.6%)
Internal mammary nodal metastasis		
Yes	11 (4.1%)	41 (15.4%)
No	8 (3.0%)	206 (77.5%)
Neck level IV nodal metastasis		
Yes	12 (4.5%)	11 (4.1%)
No	7 (2.6%)	236 (88.8%)
